# Road Proximity Increases Risk of Skeletal Abnormalities in Wood Frogs from National Wildlife Refuges in Alaska

**DOI:** 10.1289/ehp.10963

**Published:** 2008-04-21

**Authors:** Mari K. Reeves, Christine L. Dolph, Heidi Zimmer, Ronald S. Tjeerdema, Kimberly A. Trust

**Affiliations:** 1 U.S. Fish and Wildlife Service, Anchorage Fisheries and Ecological Services Office, Anchorage, Alaska, USA; 2 Ecology Graduate Group and Department of Environmental Toxicology, College of Agricultural and Environmental Sciences, University of California, Davis, Davis, California, USA; 3 Water Resources Science Program, University of Minnesota, St. Paul, Minnesota, USA; 4 Western Washington University, Huxley College of the Environment, Bellingham, Washington, USA

**Keywords:** abnormality, Alaska, amphibian, *Lithobates sylvaticus*, malformation, national wildlife refuge, *Rana sylvatica*, wood frog

## Abstract

**Background:**

Skeletal and eye abnormalities in amphibians are not well understood, and they appear to be increasing while global populations decline. Here, we present the first study of amphibian abnormalities in Alaska.

**Objective:**

In this study we investigated the relationship between anthropogenic influences and the probability of skeletal and eye abnormalities in Alaskan wood frogs (*Rana sylvatica*).

**Methods:**

From 2000 to 2006, we examined 9,269 metamorphic wood frogs from 86 breeding sites on five National Wildlife Refuges: Arctic, Innoko, Kenai, Tetlin, and Yukon Delta. Using road proximity as a proxy for human development, we tested relationships between skeletal and eye abnormalities and anthropogenic effects. We also examined a subsample of 458 frogs for the trematode parasite *Ribeiroia ondatrae*, a known cause of amphibian limb abnormalities.

**Results:**

Prevalence of skeletal and eye abnormalities at Alaskan refuges ranged from 1.5% to 7.9% and were as high as 20% at individual breeding sites. Proximity to roads increased the risk of skeletal abnormalities (*p* = 0.004) but not eye abnormalities. The only significant predictor of eye abnormalities was year sampled (*p* = 0.006). *R. ondatrae* was not detected in any Alaskan wood frogs.

**Conclusions:**

Abnormality prevalence at road-accessible sites in the Kenai and Tetlin refuges is among the highest reported in the published literature. Proximity to roads is positively correlated with risk of skeletal abnormalities in Alaskan wood frogs.

Amphibian populations, often considered sentinels of ecologic health and indicators of environmental change (Van der Schalie et al. 1999), are declining worldwide (Stuart et al. 2004). Concurrent with this decline is an apparent increase in morphologic abnormalities (Hoppe 2000). Although the background rate of abnormalities in wild amphibian populations has been described as between 0% and 5% (Converse et al. 2000; Eaton et al. 2004; Gurushankara et al. 2007; Hoppe 2000; Johnson et al. 2002; Ouellet 2000; Schoff et al. 2003; Taylor et al. 2005), recent studies of frogs in some areas have documented rates as high as 6–22% (Bacon et al. 2006; Levey 2003; McCallum and Trauth 2003). Established causes of limb abnormalities in amphibians include parasites, chemical contaminants, ultraviolet-B radiation (UVB), and invertebrate predators (Blaustein and Johnson 2003). Causes of eye abnormalities are less well understood, but authors have suggested chemical contaminants and early-season temperature extremes (Vershinin 2002) or a recessive genetic mutation (Nishioka 1977). In field studies, high abnormality prevalence has been correlated with human activities such as urbanization and agricultural and industrial land use (Gurushankara et al. 2007; Hopkins et al. 2000; Ouellet et al. 1997; Taylor et al. 2005; Vershinin 2002). In assessing current trends in environmental health, pivotal questions remain about the extent to which human activities are driving amphibian abnormalities in different parts of the world (Johnson et al. 2007; Skelly et al. 2007; Taylor et al. 2005).

The prevalence of abnormalities in Alaskan amphibians had not been examined before this study. The highest-latitude studies of this type were in central Canada (55°7′48 N; Eaton et al. 2004) and Russia (56°51′00 N; Vershinin 2002). Alaska represents an important place to examine hypotheses about amphibian abnormalities for a number of reasons. In contrast to the contiguous 48 states—where ecologic cause-and-effect relationships are confounded by multiple broad-scale land-use alterations—Alaska is characterized by vast stretches of wilderness punctuated by local and self-contained disturbances such as roads and small towns. As such, it offers a unique opportunity to isolate the effects of human activities on amphibian populations. Second, the extreme northern latitude of Alaska allows for consideration of the UVB hypothesis for limb abnormalities (Ankley et al. 2004), because long summer days increase the duration of UVB exposure during tadpole development. Finally, because Alaska contains the largest tracts of protected land in the country, it is important from a natural resource management and conservation perspective to develop a baseline understanding of amphibian health in the region.

Here, we present the first study of abnormal amphibians in Alaska: a large, systematic, multiyear sampling effort, which documents the prevalence and types of abnormalities in wood frogs (*Rana sylvatica*, also called *Lithobates sylvaticus*) from five different National Wildlife Refuges. We also analyzed the relationship between anthropogenic landscape alterations, approximated by the presence of roads, and abnormality prevalence to assess the effect of human activities on Alaskan amphibians.

## Materials and Methods

### Species, refuge, and site selection

*R. sylvatica* (Hillis 2007) is the only amphibian common in most of Alaska, and the only amphibian in the refuges we studied. Wood frogs breed explosively just after snowmelt, laying eggs in late April or early May and metamorphosing in late June or July (Herreid and Kinney 1967). After metamorphosis, young frogs migrate up to 2 km from breeding wetlands to adult habitat in adjacent woods (Berven and Grudzien 1990). This synchronous breeding and development at each site cause larvae to metamorphose within a 5- to 7-day window (Herreid and Kinney 1967; Reeves MK, unpublished data). We examined frogs for abnormalities only during this time.

**Figure 1:**
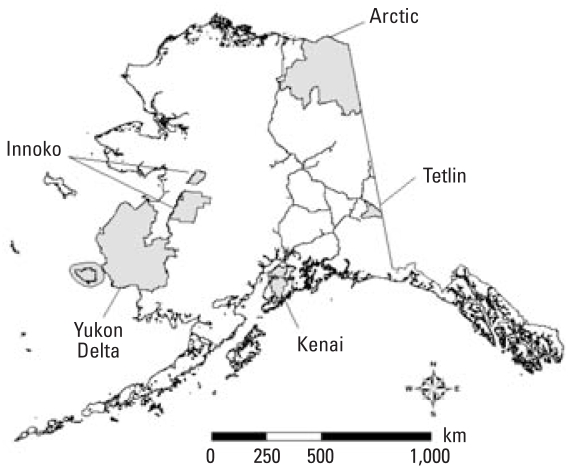
Map of Alaska showing locations of refuges sampled for abnormal wood frogs.

We chose five refuges in Alaska for this study—Arctic, Innoko, Kenai, Tetlin, and Yukon Delta (Figure 1, Table 1)—based on known frog presence and geographic location in the state. We chose sampling sites within each refuge based on proximity to roads and logistics of site access. We used geographic information systems (ESRI) and site latitude/ longitude data (World Geodetic System 1984) to calculate distance to the nearest road.

All sites in the Arctic and Innoko refuges are in remote wilderness areas, accessible only by float plane or river boat. Sites in these refuges are clustered along rivers or lakes, near permanent camps or cabins from which sampling was based.

All sites within the Yukon Delta Refuge were in the town of Bethel (population 6,262) and were accessed by road. Bethel is a shipping and transportation hub for western Alaska, but it is not on the main highway system and lacks road access to other Alaskan cities. Potential contaminant sources associated with roads in Bethel include gravel operations, landfills, sewage treatment facilities, and defunct military communications sites.

In the Kenai and Tetlin refuges, we sampled both road-accessible and wilderness sites. The Kenai Refuge has 345 km of roads, including the only major highway bisecting the Kenai Peninsula. Many of these roads were developed to support the two operating oil and gas fields in the refuge, the first of which began drilling in the 1950s. Oil and gas development and other road-associated human activities in the Kenai Refuge have led to the release of contaminants, including pentachlorophenol, petroleum products, poly-chlorinated biphenyls, mercury from historic mining, and historic herbicide applications (Parson 2001). The site farthest from any road in the Kenai Refuge is 10 km. In the Tetlin Refuge, approximately half the sites lie along the Alaska–Canada highway (the only highway connecting Alaska to the coterminous United States), and half are near Jathamund Lake, between 35 and 40 km from the nearest road. At Tetlin, former military installations, transportation corridors, and a natural gas pipeline (which parallels the highway) have all led to environmental contamination. Contaminants associated with former military activities include petroleum products and pesticides (Rocque 2007). The pipeline route was sprayed with dioxin-containing herbicides in the 1960s (Rocque 2007).

### Animal collection

Between 50 and 100 metamorphic frogs, stage 42–46 (Gosner 1960), were assessed for abnormalities at each site. Stages 42–44, which are mainly aquatic, were captured with dip nets, and stages 45–46, which are primarily terrestrial, were caught by hand at the pond edge. Frogs were placed in buckets at the capture site until they were examined for abnormalities using standard protocols [U.S. Fish and Wildlife Service (FWS) 1999]. Snout-to-vent length (SVL) and tail length were measured, and developmental stage was recorded. Abnormal frogs were euthanized with tricaine methanesulfonate (MS-222; Argent Chemical Laboratories, Redmond, WA), photographed, and sent to the U.S. Geological Survey, National Wildlife Health Center, or Ball State University for radiographs to aid in abnormality classification. A subset of normal and abnormal frogs from Kenai (*n* = 448) and Tetlin (*n* = 10) were examined for parasites, including *R. ondatrae*, at the University of Wisconsin, La Crosse. All normal frogs not collected for parasitology were released at the capture site after field examination. Equipment was disinfected with 5% bleach solution between sites to prevent disease spread. All animals were treated humanely with regard to alleviation of suffering and according to U.S. government principles for the use and care of vertebrate animals used in testing, research, and training.

### Abnormality classification

According to Johnson et al. (2001), “abnormality” is a general term referring to “any gross deviation from the normal range in morphological varition” and includes both “malformations” (permanent structural defects resulting from abnormal development) and “deformities” (alterations, such as amputation, to an otherwise correctly formed organ or structure). We categorized abnormalities for analysis using standard protocols (U.S. FWS 2007) and published guides (Meteyer 2000), and subdivided them into the following categories: skeletal abnormalities, eye abnormalities, surface abnormalities (e.g., wounds, skin discolorations, cysts), and diseases. Animals with only surface abnormalities or diseases were considered normal in this analysis. Skeletal abnormalities include three subcategories: malformations, injuries, and abnormalities of unknown origin (Table 2). A single researcher classified all frogs in this data set from pictures, radiographs, and field notes.

### Statistical analysis

To examine potential risk factors associated with abnormality prevalence in Alaskan wood frogs, we performed a regression analysis of skeletal and eye abnormalities as a function of breeding site characteristics and covariates. Explanatory variables included frog length, frog developmental stage, year the frogs were found, and refuge in which the frogs were found. We used frog length and stage as covariates; the refuge parameter represented large-scale geographic patterns; and year represented environmental variables that change annually (e.g., temperature, UVB). As a surrogate for human disturbance (chemical habitat alteration or predator, pathogen, or parasite introduction) we also included distance from breeding sites to the nearest road, which we log-transformed before analysis to make the relationship with abnormalities linear. In our study areas, distance to road is a better predictor of chemical contamination than is distance to nearest population center (Parson 2001; Rocque 2007).

We first used logistic regression with stepwise selection to identify factors that were significant predictors for each abnormality type (skeletal and eye abnormalities). We then used a generalized linear model (GENMOD in SAS, version 9.1; SAS Institute Inc., Cary, NC) to perform a repeated-measures analysis, which specified that individuals from the same collection event (animals at the same site in the same year) were correlated. This second analysis tended to reduce the significance values of factors in the original model. We dropped variables if they were significant during the stepwise selection but not significant once the repeated-measures analysis accounted for autocorrelation in the data. After we dropped nonsignificant factors, we reran the repeated-measures analysis a final time to obtain *p*-values and odds ratios (ORs).

The original sample contained 9,268 metamorphs examined between 2000 and 2006; we excluded 272 from statistical analysis because we lacked information about the site, frog length, or stage, leaving 8,997 for the skeletal abnormality and malformation analyses. We used only data from 2003–2006 for the eye abnormality analysis (*n* = 7,136) because of a change in eye abnormality protocols in 2003. We performed all analyses using SAS software. Error bars in figures were calculated based on the underlying binomial distribution


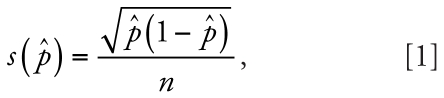


where *s*(p̂) is the standard error estimate, p̂ is the proportion abnormal in that category, and *n* is the number sampled in each category.

## Results

We examined a total of 9,268 metamorphic wood frogs from 86 breeding sites at five Alaskan refuges in this study. Abnormalities were observed at all refuges sampled. Kenai had the highest prevalence of abnormal individuals (7.9%), followed by Tetlin (5.9%), Innoko (3.0%), Arctic (2.0%), and Yukon Delta (1.5%). The overall prevalence of abnormal frogs was 6.2%.

The highest abnormality prevalence at any single breeding site was 20%, at a Kenai site in 2005 (Table 2). In Innoko, the highest single-site abnormality prevalence was 19%; in Tetlin, 14%; Arctic, 6%; and Yukon Delta, 5%. Each refuge had sites at which no abnormal frogs were found, but this was not the norm; 88% of the 161 sampling events yielded at least one abnormal frog.

**Figure 2:**
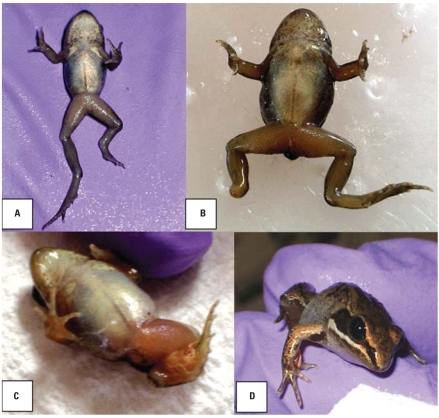
The four most common abnormalities in Alaskan wood frogs: (*A*) micromelia, (*B*) ectromelia, (*C*) amelia, and (*D*) unpigmented iris.

More than 20 different types of abnormalities were documented (Table 2). Ectromelia (partial limb), micromelia (shrunken limb or limb element), amelia (limb totally missing), and unpigmented iris (eye totally black) were the four most common, collectively accounting for 73% of the abnormalities across all refuges (Figure 2; Table 2). These abnormalities were also the most common at each individual refuge, with some exceptions. Black-eyed frogs were common at Innoko, Kenai, and Tetlin, making up ≥ 20% of the abnormalities at each refuge, but only one black-eyed frog was found in Yukon Delta, and none were found in Arctic. Shrunken and partial limbs were among the most common abnormality types at all refuges except Innoko, which had a high proportion of partial limbs (27% of the abnormalities were of this type) but fewer shrunken limbs (only 7% of the abnormalities at this refuge). Several abnormality types occurred only in Kenai, including anteversion (twisted long bones), microcephaly (shrunken head), scoliosis (curved spine), cutaneous fusion (skin webbing), and kinked tail (Table 2). The rarest abnormality type was polymelia (extra limb); only one specimen had an extra limb, and this frog was also found in Kenai. Injuries comprised 12–36% of the skeletal abnormalities at each refuge, with the lowest proportion at Innoko, the highest at Yukon Delta, and more intermediate values at Arctic (17%), Kenai (17%), and Tetlin (20%).

The trematode parasite *R. ondatrae* is known to induce skeletal malformations in amphibians (Johnson and Sutherland 2003). To investigate whether *R. ondatrae* could be implicated in the abnormalities identified, we kept a subset of normal and abnormal frogs collected in the field for parasite analysis. We examined a total of 448 specimens from Kenai and 10 from Tetlin for parasites. None of these frogs were infected with *R. ondatrae*, nor were planorbid snail hosts seen at any sampling site.

**Figure 3:**
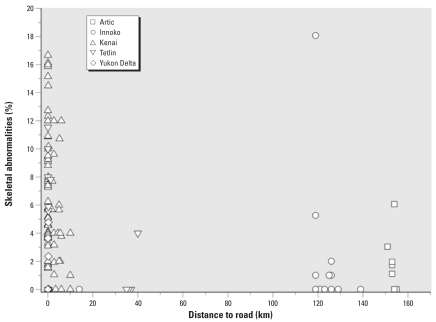
Skeletal abnormalities and malformations versus distance to the nearest road. Symbols indicate prevalence of frogs with skeletal abnormalities during single collection events at different refuges.

**Figure 4:**
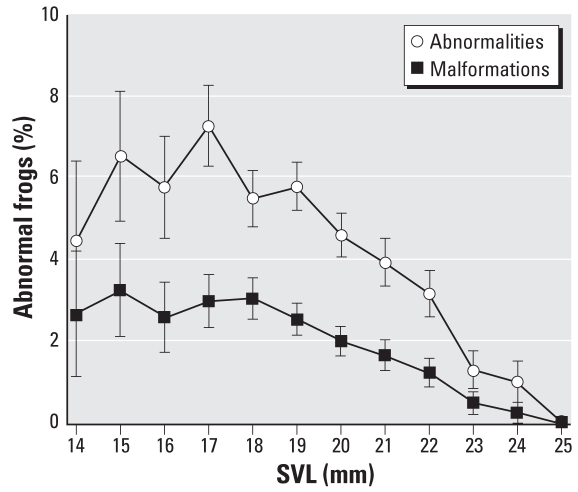
Skeletal abnormalities and malformations shown as the proportion of abnormal frogs at each SVL (mean ± SE, where SE is based on Equation 1).

**Figure 5:**
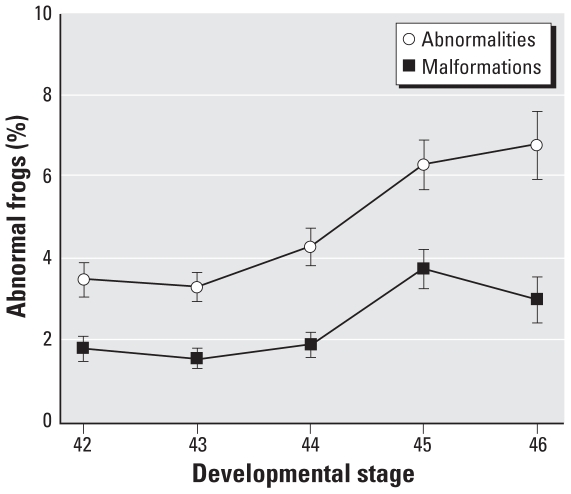
Skeletal abnormalities and malformations shown as the proportion of abnormal frogs at each developmental stage (mean ± SE, where SE is based on Equation 1).

In the regression analysis, prevalence of skeletal abnormalities increased with site proximity to the nearest road [*p* = 0.004, odds ratio (OR) = 0.8809; Figure 3). With one exception, all sites with abnormality prevalence > 6% were within 10 km of a road. One remote site in Innoko deviated from this trend (shown as an outlier in Figure 3, with an abnormality prevalence of 19%). This site, located > 100 km from any road, is adjacent to a historic mining and trapping cabin, now used as the base of Innoko Refuge field operations. This outlier did not affect our result interpretation, so we retained it during statistical analysis. Frogs in our study were also more likely to have skeletal abnormalities if they were smaller (*p* = 0.002, OR = 0.8831; Figure 4) and at a later developmental stage (*p* < 0.0001, OR = 1.2812; Figure 5). The preliminary logistic regression analysis identified significant differences in skeletal abnormalities among refuges; however, once we accounted for autocorrelation in our data with the repeated-measures analysis, refuge was no longer a significant predictor of any abnormality type. We found no relationship between skeletal abnormalities and year sampled. Eye abnormalities varied with year (*p* = 0.006), although they were not correlated with refuge, frog size, Gosner stage, or distance to the nearest road. Significantly fewer eye abnormalities were found in 2003 than in 2004 (OR = 0.1969) or 2005 (OR = 0.2078).

In our data, frogs found closer to roads were smaller. In a simple linear regression of frog size against distance to the nearest road, the equation is SVL (millimeters) = 19.2 + [0.02 × distance to road (kilometers)] (*p* < 0.0001; *R*^2^ = 0.20). By this equation, the average SVL of frogs in a site adjacent to the road is 19 mm, whereas the average SVL at 150 km is 22 mm. Despite this collinearity between size and distance to roads, we included both in our final regression model because both were significant during stepwise model selection, suggesting this collinearity was overcome. Additionally, both factors could independently influence abnormality prevalence, so we avoided choosing one or the other to represent both.

## Discussion

The average abnormality prevalence in this study (6.2%) is higher than background levels of 0–5% reported for other areas (Ouellet 2000). The average in this study is high, however, because of hotspots in some areas. Specifically, the abnormality prevalence at road-accessible sites in the Kenai and Tetlin refuges is among the highest reported to date. Remote areas in Alaskan refuges exhibited abnormality prevalence closer to 2% and within the published range for background levels in other places in North America (Converse et al. 2000; Eaton et al. 2004; Hoppe 2000; Schoff et al. 2003; Taylor et al. 2005).

We observed higher abnormality prevalence in sites closer to roads. Ostensibly, road proximity could increase the prevalence of frog abnormalities by contributing to chemical contamination of the habitat (Parson 2001; Rocque 2007) or by facilitating introduction of predators, parasites, or pathogens (Reeves 2008; Urban 2006). If contaminants caused abnormal development in Alaskan amphibians, then proximity to roads should result in malformations but not injuries in the absence of other stressors (Loeffler et al. 2001). If predators caused the limb abnormalities, we should see fresh and healed injuries and possibly developmental malformations if limbs were amputated early enough in tadpole development to partially regenerate (Forsyth 1946; Fry 1966). The prevalence of both malformations and injuries in our data suggests that predators were almost certainly responsible for some proportion of the skeletal abnormalities. Either early limb amputation by predators (Forsyth 1946; Fry 1966) or exposure to chemical contaminants (Gardiner et al. 2003) may have caused the developmental malformations. Road-associated contaminants may also reduce tadpole size or fitness, increasing the risk of predation injury (Boone and James 2003). Nevertheless, both chemical contaminants (Relyea 2005) and invertebrate predators (Relyea 2001) can decrease frog size at metamorphosis. Thus, we cannot discern whether road effects on skeletal abnormalities are mediated through chemical contaminants, shifts in predator community composition, or a combination of these two stressors.

Our data do not support the parasite or UVB hypotheses for skeletal abnormalities in Alaskan wood frogs. We did not detect the malformation-inducing parasite *R. ondatrae* in any of the frogs in this study, and the lack of bilateral malformations is atypical of UVB exposure (Ankley et al. 2002). It is possible that UVB induced the eye abnormalities in our study, based on the correlation with year sampled, yet we cannot find any report that associates UVB with amphibian eye abnormalities. Causes of eye abnormalities in amphibians are not well understood, but others have proposed chemical contaminants, temperature extremes, and genetic mutations as causes (Nishioka 1977; Vershinin 2002). We cannot rule out temperature extremes or genetic mutations as causes of the eye abnormalities in this study, but chemical contaminants are unlikely candidates, based on the lack of correlation with roads and associated environmental contamination.

A number of limitations are associated with using road proximity as the only means by which to quantify the effects of human disturbance on Alaskan wood frogs. For example, not all roads in this study represent the same kind of landscape disturbance. Whereas all of the Yukon Delta sites were closest to roads in the town of Bethel, a small village accessible only by air or barge, in Kenai the nearest road may have been either a major highway or a restricted-access gravel road on the oil and gas fields. Moreover, roads are not necessarily the only or even the most significant source of human disturbance to a breeding site. The high abnormality prevalence at one remote Innoko site may be an example of anthropogenic effects unrelated to roads, because this site was subject to stressors related to current refuge operations and historic land use. Clearly, further study is needed to discern whether and how human activities are related to abnormalities in Alaskan frogs.

In addition to the correlation between road proximity and abnormality prevalence, we identified other significant covariates, including frog size, frog developmental stage, and year sampled. Several mechanisms could explain the increased probability of skeletal abnormalities with smaller size. Small frogs might be more likely to suffer insults such as failed predation attempts. Size at metamorphosis has been related to adult fitness (Werner 1986), and small tadpoles and metamorphs are more vulnerable to gape-limited predation (Brodie and Formanowicz 1983). Alternatively, abnormal frogs may compete poorly for resources, leaving them smaller at metamorphosis than their normal counterparts. Finally, the stressor causing abnormalities could also reduce size at metamorphosis. Wood frogs exposed to caged predators (Relyea 2001) and chemical contaminants (Relyea 2005) were smaller at metamorphosis than unexposed controls. Size and developmental stage were not correlated in our data.

The increased prevalence of skeletal abnormalities at later developmental stages is probably sampling bias created by different capture techniques. Whereas dip netting for earlier-stage metamorphs (Gosner stage 42–44) samples abnormal and normal individuals with comparable efficiency, capturing later-stage metamorphs on land may result in the disproportionate collection of the less-mobile abnormal animals. Moreover, normal metamorphs leave the breeding area quickly, but frogs with skeletal abnormalities may stay closer to water, where they can dive from predators instead of relying on missing or misshapen limbs to escape. Care was taken to examine each limb during sampling, because the primary goal of this study was detection of morphologic abnormalities. Therefore, we do not think limb abnormalities were obscured by the longer tails of earlier-stage metamorphs (another potential source of sampling bias).

Correlative models provide results valuable for focusing future data collection. Our model identified contaminants and predators, or a synergistic interaction between them, as important areas of future research into the causes of limb abnormalities in Alaskan wood frogs. Our data also suggest that *R. ondatrae* and UVB are probably not responsible for the skeletal abnormalities we observed, but UVB or climate may cause the eye abnormalities.

## Conclusion

The elevated abnormality prevalence in some areas of Alaska’s National Wildlife Refuges is a striking indication that we cannot assume the size and relative remoteness of these protected areas render them immune to the influence of humans. On the other hand, although preliminary evidence points to a possible effect of anthropogenic disturbance on Alaskan wood frogs, we lack sufficient evidence to identify a specific causal agent. The results of our analyses suggest that predation injuries and some effect of roads, such as chemical contamination or shifts in predator community composition, may contribute to the skeletal abnormalities we observed. The cause of eye abnormalities is unknown, yet the lack of association with human disturbance and the significance of year sampled in our statistical model suggest that eye abnormalities in Alaskan wood frogs are more likely to be associated with something that occurs statewide and changes annually, such as UVB or climate. More study is needed to elucidate risk factors for amphibian abnormalities in Alaska, and such research is ongoing.

## Figures and Tables

**Table 1:** Skeletal and eye abnormality and breeding site information by refuge.

Refuge	Years sampled	No.sites^b^	Abnormalities (%)^a^			Latitude	Longitude	Site elevation (m)	Distance to road (km)
			Mean	Median	Range				
Arctic (7,932,000 ha)	2001–2002	9	2.0	1.4	0–6	67°10′48″–67°13′12″N	142°7′48″–142°11′59″W	195–200	151–155
Innoko (1,558,000 ha)	2002–2006	13	3.0	1.5	0–19	63°36′0″–63°38′24″N	158°1′48″–158°8′24″W	25–30	14–139
Kenai (797,200 ha)	2000–2006	38	7.9	7.6	0–20	60°8′42″–60°46′48″N	150°3′36″–151°5′24″W	60–520	0–10
Tetlin (95,426 ha)	2003–2006	19	5.9	4.0	0–14	62°38′24″–62°58′12″N	141°1′48″–141°51′36″W	500–700	0–40
Yukon Delta (6,555,850 ha)	2002–2004	7	1.5	0.0	0–5	60°46′48″–60°47′24″N	161°48′36″–161°52′48″W	15–30	0–5

Data for site latitude and longitude from WGS (1984).

a Mean refuge overall abnormality prevalence = number abnormal frogs/total frogs sampled at all sites over all years.

b Number of breeding sites sampled.

c For the median and range of breeding site abnormality prevalence we did not calculate prevalence of skeletal and eye abnormalities for ponds at which < 50 individuals were examined.

**Table 2:** Summary of abnormalities in wood frog populations at five national wildlife refuges in Alaska.

	No. of abnormalities					
Abnormality	Arctic	Innoko	Kenai	Tetlin	Yukon Delta	Total
Eye abnormality						
Anophthalmia (missing eye)	0	2	12	6	0	20
Unpigmented iris (black eye)	0	15	118	20	1	154
Microphthalmia (small eye)	0	0	1	1	0	2
Other^a^	0	2	6	2	0	10
Skeletal injury^b^						
Brachydactyly (short digits)	2	0	7	0	2	11
Ectrodactyly (missing digits)	1	1	4	3	0	9
Ectromelia (partial limb)	0	0	44	6	0	50
Limb crushed	0	0	14	1	2	17
Other^c^	0	2	2	0	0	4
Skeletal malformation						
Amelia (missing limb)	0	1	31	3	0	35
Anteversion (twisted long bones)	0	0	9	0	0	9
Brachygnathia (short jaw)	1	4	6	0	0	11
Microcephaly (shrunken head or blunt snout)	0	0	4	0	0	4
Micromelia (shrunken limb or limb element)	5	3	126	17	3	154
Polymelia (extra limb)	0	0	1	0	0	1
Polydactyly (extra digits)	2	0	2	0	0	4
Scoliosis or lordosis (curved spine)	0	0	2	0	0	2
Cutaneous fusion (skin webbing)	0	0	3	0	0	3
Syndactyly (digits fused)	0	0	11	2	0	13
Taumelia (bone bridge or triangle)	0	0	4	0	0	4
Skeletal unknown origin						
Kinked tail	0	0	3	0	0	3
Brachydactyly (short digits)	0	1	27	1	2	31
Ectrodactyly (missing digits)	0	0	26	3	0	29
Ectromelia (partial limb)	7	12	90	14	2	125
Other^d^	0	2	5	0	0	7
Overall						
Eye total	0	19	137	29	1	186
Injury total	3	3	71	10	4	91
Malformation total	8	8	199	22	3	240
Unknown origin total	7	15	151	18	4	195
Total abnormalities	18	45	558	79	12	712
Total abnormal individuals	12	39	450	68	7	576
Total individuals examined	615	1,309	5,716	1,146	482	9,268
Percent individuals abnormal	2.0%	3.0%	7.9%	5.9%	1.5%	6.2%

a Includes oversized eyes, abnormally shaped pupils, and cataracts.

b Either fresh blood or exposed bone was noted for the injury category.

c Includes dissociated and dangling limb.

d Includes apparent dislocations.
